# CO_2_ Activation and Hydrogenation on Cu-ZnO/Al_2_O_3_ Nanorod Catalysts: An In Situ FTIR Study

**DOI:** 10.3390/nano12152527

**Published:** 2022-07-23

**Authors:** Letian Wang, Ubong Jerome Etim, Chenchen Zhang, Lilac Amirav, Ziyi Zhong

**Affiliations:** 1 Department of Chemical Engineering, Guangdong Technion-Israel Institute of Technology (GTIIT), Shantou 515063, China; wang.letian@gtiit.edu.cn (L.W.); ubong.etim@gtiit.edu.cn (U.J.E.); chenchen.zhang@gtiit.edu.cn (C.Z.); 2 Schulich Faculty of Chemistry, Technion-Israel Institute of Technology (IIT), Haifa 32000, Israel; 3 Guangdong Provincial Key Laboratory of Materials and Technologies for Energy Conversion (MATEC), Guangdong Technion-Israel Institute of Technology (GTIIT), Shantou 515063, China

**Keywords:** CO_2_ adsorption, CO_2_ activation, CO_2_ hydrogenation, CuZnO/Al_2_O_3_, catalyst components

## Abstract

CuZnO/Al_2_O_3_ is the industrial catalyst used for methanol synthesis from syngas (CO + H_2_) and is also promising for the hydrogenation of CO_2_ to methanol. In this work, we synthesized Al_2_O_3_ nanorods (n-Al_2_O_3_) and impregnated them with the CuZnO component. The catalysts were evaluated for the hydrogenation of CO_2_ to methanol in a fixed-bed reactor. The support and the catalysts were characterized, including via in situ diffuse reflectance infrared Fourier transform spectroscopy (DRIFTS). The study of the CO_2_ adsorption, activation, and hydrogenation using in situ DRIFT spectroscopy revealed the different roles of the catalyst components. CO_2_ mainly adsorbed on the n-Al_2_O_3_ support, forming carbonate species. Cu was found to facilitate H_2_ dissociation and further reacted with the adsorbed carbonates on the n-Al_2_O_3_ support, transforming them to formate or additional intermediates. Like the n-Al_2_O_3_ support, the ZnO component contributed to improving the CO_2_ adsorption, facilitating the formation of more carbonate species on the catalyst surface and enhancing the efficiency of the CO_2_ activation and hydrogenation into methanol. The synergistic interaction between Cu and ZnO was found to be essential to increase the space–time yield (STY) of methanol but not to improve the selectivity. The 3% CuZnO/n-Al_2_O_3_ displayed improved catalytic performance compared to 3% Cu/n-Al_2_O_3_, reaching a CO_2_ conversion rate of 19.8% and methanol STY rate of 1.31 mmolg_cat_^−1^h^−1^ at 300 °C. This study provides fundamental and new insights into the distinctive roles of the different components of commercial methanol synthesis catalysts.

## 1. Introduction

In the last two decades, global warming and related extreme weather conditions have become topical issues, and the mitigation of carbon dioxide (CO_2_) release into the atmosphere is a top global priority. CO_2_ is a major anthropogenic greenhouse gas (GHG) released during fossil fuel exploitation; thus, controlling CO_2_ emissions is critical to maintaining the carbon-neutral state of the atmosphere [1,2,3]. The catalytic reduction of CO_2_ with renewable hydrogen (H_2_) to value-added chemicals and fuels such as hydrocarbons and alcohols represents a potential strategy to mitigate CO_2_ emissions into the atmosphere [1,4,5,6]. For example, the hydrogenation of CO_2_ to methanol is an important reaction in CO_2_ conversion, as methanol can be used as a feedstock chemical and fuel [7]. However, CO_2_ is a thermodynamically inert molecule that needs high reaction temperatures to activate the C=O bond. At high temperatures, the undesired reverse water–gas shift (RWGS) reaction is thermodynamically favored, which reduces the product selectivity. Therefore, developing a catalyst that can efficiently drive the process under mild conditions is imperative.

Different types of catalysts have been investigated for the thermocatalytic hydrogenation of CO_2_ to methanol, including supported metal [8,9] and metal oxide [10,11] catalysts. Cu-based catalysts have been reported to have the best activity for methanol production under industrially relevant conditions (5–10 MPa and 200–300 °C) [12,13]. The Cu-ZnO-Al_2_O_3_ catalyst prepared via the co-precipitation method is the industrial catalyst used for methanol production from CO + H_2_; thus, the Cu-ZnO-based catalysts are widely investigated for CO_2_ hydrogenation to methanol as well [14,15]. Various supports and promoters are explored for these catalysts, and different catalyst structures and active sites are reported [15,16,17]. The common supports or promoters for Cu-based catalysts include ZnO, ZnO-Al_2_O_3_, ZrO_2_, TiO_2_, In_2_O_3_, CeO_2_, and SiO_2_ [18,19]. At times the contribution of the support is complex. For example, Nitta et al. [18] observed significantly improved selectivity towards methanol formation at a low temperature upon introducing ZnO into Cu/ZrO_2_. However, at high temperatures, adding ZnO promoted the methanol decomposition into CO, leading to a reduced yield for methanol.

Factors impacting the overall catalytic performance of the Cu-based catalysts include the nature of the support material, the Cu loading, the dispersion, and the preparation method [12,20]. However, their effects on the activation of CO_2_ are still not clear. The specific roles of the different components within the industrial Cu-ZnO-Al_2_O_3_ catalyst for methanol synthesis from CO_2_ were assigned in the literature [12,18]. ZnO is generally believed to be a multitasked component, acting as a physical spacer for the Cu particles and facilitating H_2_ dissociation; Al_2_O_3_ plays the role of a structural promoter that increases Cu dispersion, whereas Cu drives the selectivity towards methanol [21,22]. However, further investigation is required to ascertain the individual roles of the catalyst components concerning CO_2_ activation, as well as to better understand the observed synergetic effects within the complete system. In addition to the composition, the nature of the catalyst support and characteristics such as the morphology (size and shape), were also found to influence the CO_2_ conversion efficiency and selectivity towards methanol formation [23,24,25,26]. An et al. [27] found that the catalytic activities of Cu/Zn/Al/Zr catalysts depended strongly on the morphology of the support, and the utilization of a fibrous shape improved the hydrogenation performance significantly. In addition, the proper selection of the catalyst support may provide additional advantages, such as reducing the amount of required active metal, inhibiting the sintering of the active metal, and prolonging the stability [22].

Diffuse reflectance infrared Fourier transform spectroscopy (DRIFTS) is a powerful technique for analyzing in situ substrate or adsorbate interactions, and has been previously utilized to characterize systems where the interactions of gases over the surface of a catalyst elucidated the catalytic surface chemistry [28,29,30,31].

In this work, we explored porous Al_2_O_3_ nanorods as the catalyst support in the CuZnO/Al_2_O_3_ catalysts for CO_2_ hydrogenation. The Al_2_O_3_ nanorods were synthesized via the steam-assisted solid wet–gel method and deployed as a support for CuZnO. The CuZnO/Al_2_O_3_ catalyst was prepared using the incipient wetness impregnation method and evaluated for the hydrogenation of CO_2_ to methanol. The catalysts supported on alumina nanorods exhibited better CO_2_ reduction to methanol under the examined conditions than that supported on commercial alumina. Through a DRIFTS study, we gained a new understanding of the specific roles of Al_2_O_3_, ZnO, and Cu in CO_2_ adsorption, activation, and hydrogenation reaction. This information will contribute to the design of catalysts with improved performance for industrial applications.

## 2. Experimental Section

### 2.1. Sample Preparation

(i) Nanorods Al_2_O_3_ preparation.

Al_2_O_3_ nanorods were synthesized using the steam-assisted solid wet–gel method according to the literature [32]. Typically, 30 g Al(NO_3_)_3_·9H_2_O (>99.9%, SCR, Sinopharm Chemical Reagent Co., Ltd., Shanghai, China) was dissolved in 50 mL of deionized water. Approximately 15% NH_4_OH aqueous solution (36%, SCR, Sinopharm Chemical Reagent Co., Ltd., Shanghai, China) was added dropwise to the aluminum nitrate solution at room temperature under stirring to control the pH at 5.0. The resulting solid precipitate was recovered by filtration. The as-prepared solid cake-like wet gel was transferred to a glass beaker (25 mL) sitting in a Teflon vessel (200 mL), where 2 g of water was poured into the bottom of the vessel but was physically separated from the solid gel sample. The Teflon vessel was sealed and heated at 200 °C for 48 h. The obtained white solid material was washed with deionized water and recovered by centrifugation, dried at 60 °C for 24 h, and subsequently calcined in air at 600 °C for 5 h to obtain Al_2_O_3_ nanorods.

(ii) Catalyst preparation.

The Al_2_O_3_-supported CuZnO catalysts were prepared via the incipient wetness impregnation method using Cu(NO_3_)_2_·6H_2_O and Zn(NO_3_)_2_·6H_2_O (>99.9%, SCR, Sinopharm Chemical Reagent Co., Ltd., Shanghai, China) (mass ratio of Cu: ZnO = 2:1) as the Cu and Zn precursors. The catalysts are labeled here as CuZnO/n-Al_2_O_3_ and CuZnO/c-Al_2_O_3_, where n-Al_2_O_3_ and c-Al_2_O_3_ are Al_2_O_3_ nanorods and commercial Al_2_O_3_ (γ-Al_2_O_3_, >99.9%, Shanghai Macklin Biochemical Co., Ltd., Shanghai, China), respectively. The loading of the active component is referred to as the total weight loading of Cu and ZnO (Cu+ZnO) in the catalysts. The resulting samples were dried overnight at 60 °C and subsequently calcined in air at 400 °C for 4 h. Catalysts with Cu+ZnO loading of 3–20% and 3% Cu were studied.

### 2.2. Characterization

The crystallinity of the samples was characterized via X-ray diffraction (XRD) experiments performed on a Bruker D8 Advance X-ray diffractometer (Cu Kα) (Bruker Corporation Inc., Billerica, MA, USA). The diffraction patterns were acquired at a scanning step of 0.01° and a scanning speed of 10°/min. Transmission electron microscopy (TEM) and scanning electron microscopy (SEM) images of the samples were recorded on a Talos F200X transmission electron microscope (Thermo Fisher Scientific Inc., Waltham, MA, USA) operated at 300 kV and a scanning electron microscope (GeminiSEM 450, Carl ZEISS, Oberkochen, Germany), respectively.

In situ DRIFTS measurements were performed on a Nicolet iS50 FTIR instrument (Thermo Fisher Scientific Inc., Waltham, MA, USA). The scans were recorded from 4000 to 600 cm^−1^. Here, 50 mg catalyst powder was placed in a high-pressure (0–10 MPa) DRIFT cell (HVC-DRP-5, Harrick Scientific Products Inc., NY, USA) equipped with ZnSe windows and reduced at 300 °C in a pure H_2_ stream (30 mL/min) for 2 h, after which the sample was flushed with N_2_ (40 mL/min) for 1 h and cooled to room temperature. The background subtractions were executed over different samples for testing in 40 mL/min N_2_ under 2 MPa. Then, the reactant gas mixture (H_2_ + CO_2_, 3:1 ratio) at a flow rate of 40 mL/min was introduced into the sample cell and the spectra were collected at different temperatures, increasing by 3 °C/min at an interval of 19 s. For the CO_2_ activation study, the sample cell was cooled to 250 °C. The background subtractions were similarly performed under 2 MPa. The CO_2_ was switched into the reaction chamber and the spectra were recorded for up to 120 min, after which the inlet gas was changed to H_2_ and the process lasted for another 1 h 40 min.

### 2.3. Catalytic Performance Evaluation

The CO_2_ hydrogenation to methanol was performed in a high-pressure fixed-bed flow reactor. Here, 0.1 g catalyst was fixed in a quartz tube using quartz wool and then packed into a stainless steel tubular reactor. Prior to the catalytic measurements, the catalyst was reduced in a gas stream (10% H_2_/N_2_) at 300 °C for 2 h with a flow rate of 10 mL/min under atmospheric pressure. Then, the temperature was decreased to 200 °C and the reducing gas was replaced by reaction gas (CO_2_ + H_2_, 1:3 ratio). The reaction was *X(CO_2_)* gas hourly space velocity (GHSV) of 7800 mL g^−1^ h^−1^. The products flowing out from the reactor passed through a tube connected to a temperature control box to maintain the temperature at 120 °C and were then analyzed using an online gas chromatographer (Shimadzu GC-2014C) equipped with a TCD and an FID. The data obtained from the GC measurements were used to calculate the CO_2_ conversion (*X(CO_2_)*), CH_3_OH selectivity (*S_i_*), and methanol space–time yield (*STY_CH3OH_*) using Equations (1)–(3).

(1) $$X\left(CO_{2}\right)=\frac{n_{co_{2},in}-n_{co_{2},out}}{n_{co_{2},in}}\times 100\%$$

(2) $$S_{i}=\frac{n_{products,\;i}}{n_{co_{2},in}-n_{co_{2},out}}\times 100\%$$

(3) $$STY_{CH3OH}=\frac{GHSV\times 0.25}{22.4}\times \frac{X\left(CO_{2}\right)S_{i}}{10000}$$

## 3. Results and Discussion

### 3.1. Catalyst Structure, Morphology and Textural Properties

**Figure 1:**
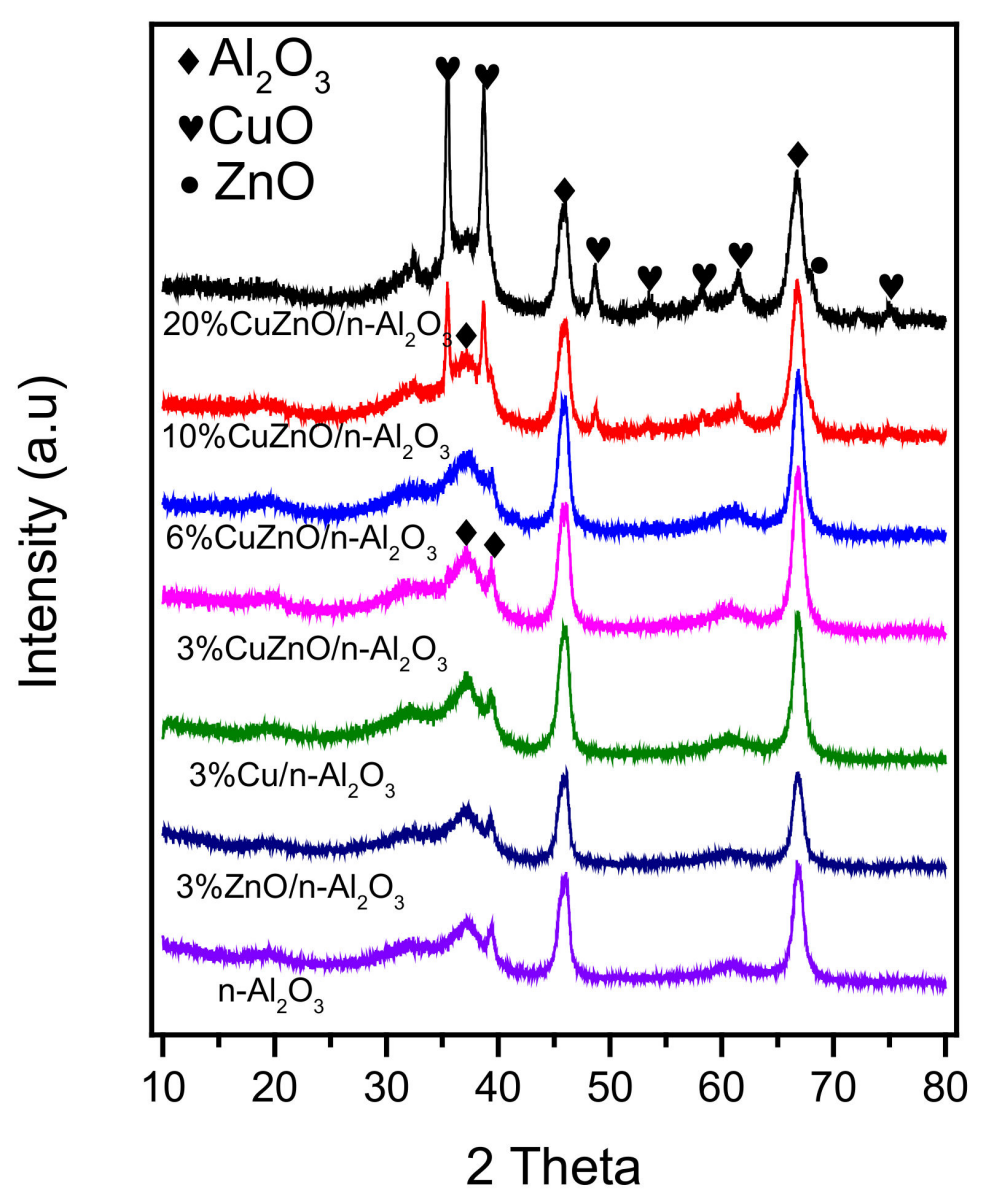
XRD patterns of n-Al_2_O_3_ and catalyst samples.

The XRD patterns of the n-Al_2_O_3_ support with various levels of CuZnO loadings are presented in Figure 1. The pattern of the catalyst support shows diffractions at the 2θ values of around 37.3, 39.4, 46.0, and 67.0°, characteristic of the γ-Al_2_O_3_ (JCPDS 04-0858). The patterns of calcined catalyst samples at low loadings (3% and 6% CuZnO) are quite similar to that of the n-Al_2_O_3_ support. No XRD peaks of CuO or ZnO can be observed, suggesting the high dispersion of CuO and ZnO, or possibly because the amount yielded in the XRD signal was close to the detection limit. In contrast to the observations for low loadings, prominent CuO XRD peaks can be observed for 10% and 20% CuZnO loadings at 35.7°, 38.6°, and 48.7° (JCPDS 80-1268).

**Figure 2:**
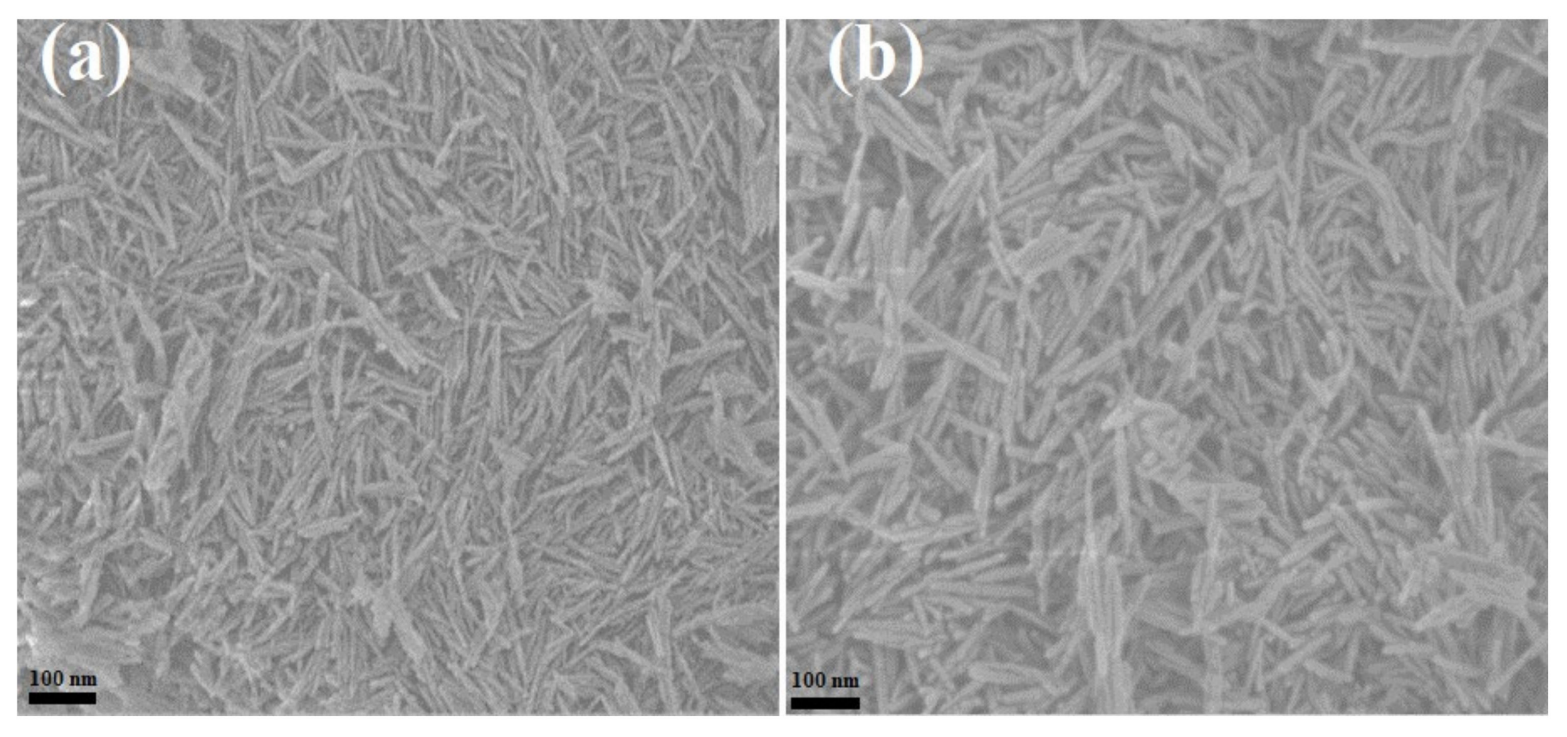
SEM images of (**a**) n-Al_2_O_3_ and (**b**) 3% CuZnO/n-Al_2_O_3_.

**Figure 3:**
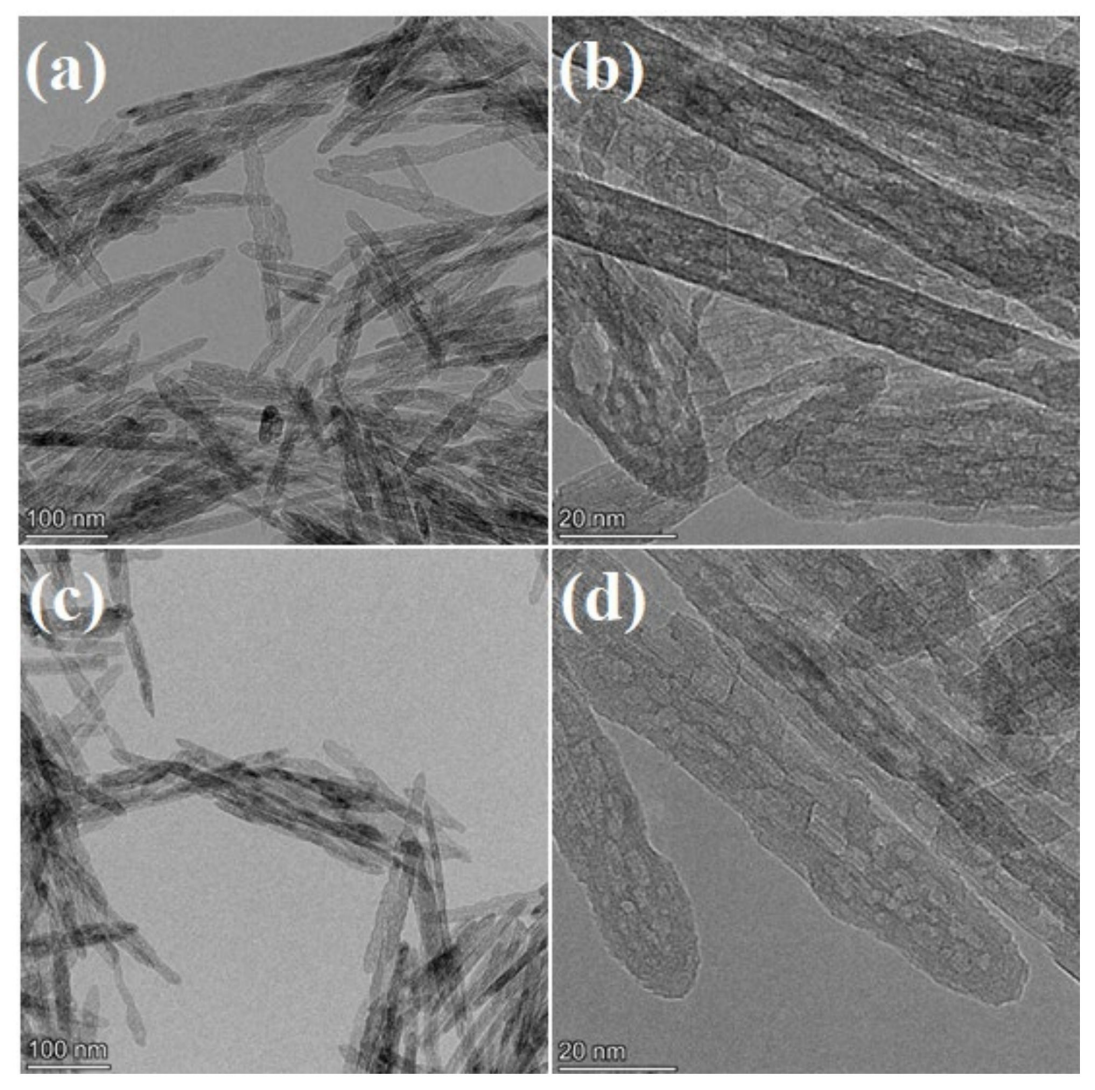
TEM images of (**a**,**b**) n-Al_2_O_3_ and (**c**,**d**) 3% CuZnO/n-Al_2_O_3_.

**Figure 4:**
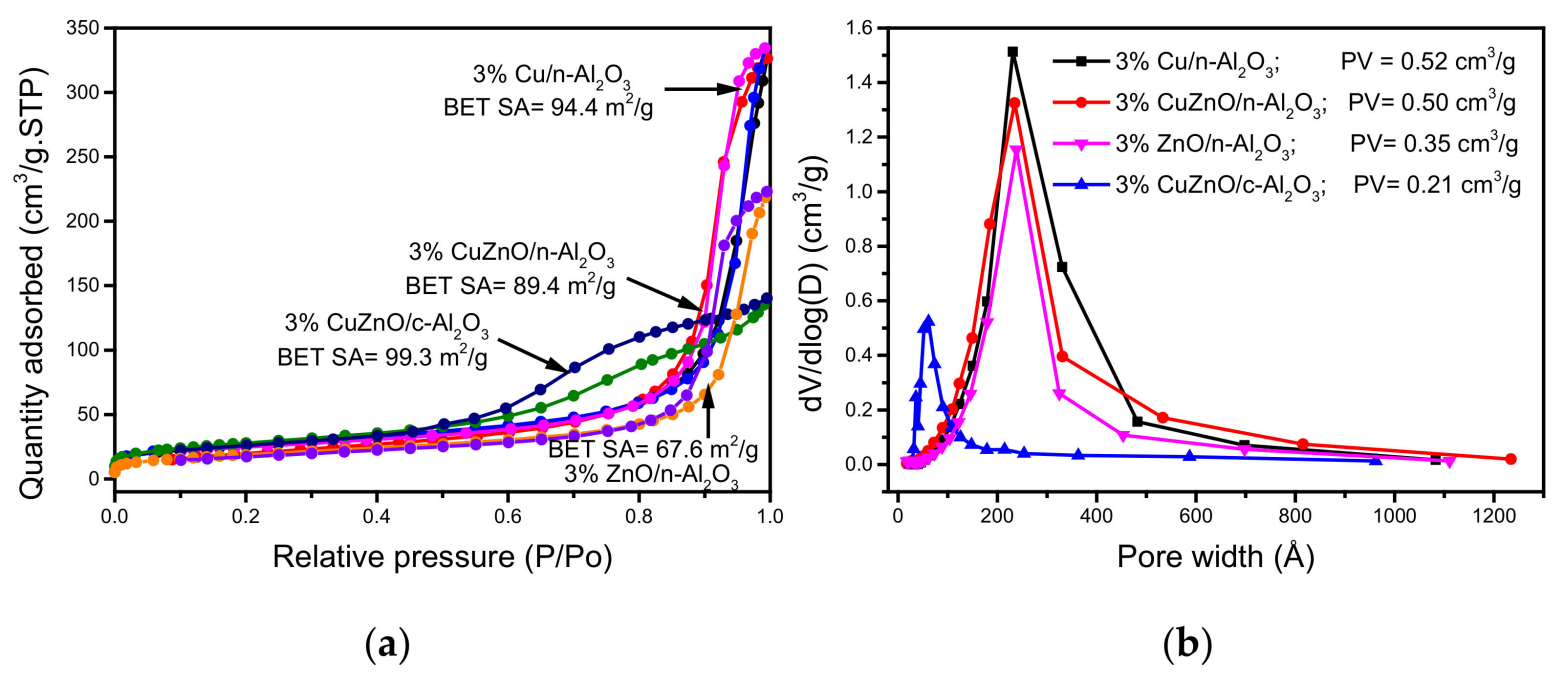
(**a**) Nitrogen adsorption–desorption isotherms and (**b**) pore size distribution curves of sample catalysts.

Figure 2a shows the morphology of the n-Al_2_O_3_ support, which can be described as γ-Al_2_O_3_ nanorods with lengths and widths of around 250–300 nm and 15–25 nm, respectively, in comparison with the spherical nano-sized particles of c-Al_2_O_3_ (figure not shown). The observed morphology is similar to that of the alumina nanorods reported in the literature [32,33]. Figure 2b shows the morphology of the n-Al_2_O_3_ impregnated with 3% CuZnO, and the corresponding EDX result presented in Table 1 shows a Cu/ZnO ratio of 2.25, which is reasonably close to the theoretical value of 2. The TEM micrographs of the n-Al_2_O_3_ and 3% CuZnO/n-Al_2_O_3_ catalysts are shown in Figure 3. After impregnating the n-Al_2_O_3_ support with 3% CuZnO, the morphology and structure of n-Al_2_O_3_ remained unchanged. Both materials consist of nanorods with pores of about 3–10 nm in diameter. Due to their low loadings and low contrast, it is difficult to visualize the CuO and ZnO particles on the surface or inside the pores of the alumina nanorods with traditional wide-field TEM [34]. In Figure 4a, the linear isotherms of Cu/n-Al_2_O_3_, CuZnO/n-Al_2_O_3_, ZnO/n-Al_2_O_3_, and CuZnO/c-Al_2_O_3_ with corresponding BET surface areas of 94.4, 89.4, 67.6, and 99.3 m^2^/g, respectively, are shown. Except for CuZnO/c-Al_2_O_3_, other samples exhibit type IV isotherms with H1-type hysteresis loops, suggesting a typical mesoporous structure related to the n-Al_2_O_3_ support [35]. CuZnO/c-Al_2_O_3_ exhibits a type II isotherm with H4-type hysteresis loops, indicating an otherwise smaller mesoporous structure. According to the PSD curves (Figure 4b), CuZnO/c-Al_2_O_3_ possesses pores exhibiting a broad range but with an average pore width of 68 Å. Despite the n-Al_2_O_3_-supported samples exhibiting a wider pore size distribution, more of the pores are concentrated in the low-diameter region up to 150 Å. Cu/n-Al_2_O_3_ and CuZnO/n-Al_2_O_3_ have a nearly similar total pore volume of approximately 0.5 cm^3^/g, which is greater than those of ZnO/n-Al_2_O_3_ and CuZnO/c-Al_2_O_3_, 0.35 and 0.21 cm^3^/g, respectively.

### 3.2. Catalytic Performance Evaluation

#### 3.2.1. CO_2_ Hydrogenation to Methanol

**Figure 5:**
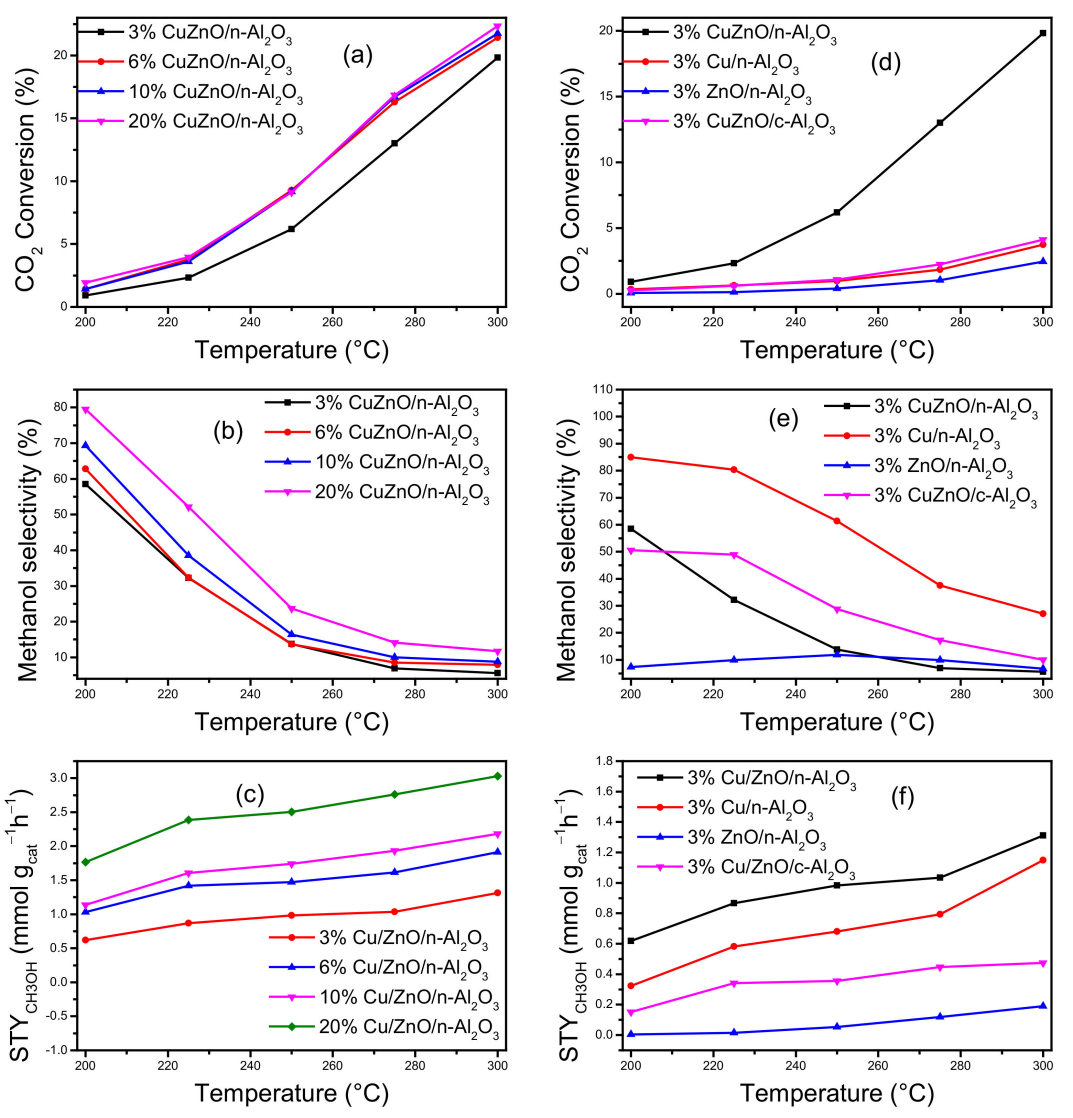
CO_2_ hydrogenation performances of catalyst samples. (**a**) CO_2_ conversion, (**b**) CH_3_OH selectivity, and (**c**) *STY_CH3OH_* of CuZnO with various loadings, supported on n-Al_2_O_3_. (**d**) CO_2_ conversion, (**e**) CH_3_OH selectivity, and (**f**) *STY_CH3OH_* of 3% Cu, 3% ZnO, and 3% CuZnO on n-Al_2_O_3,_ and 3% CuZnO on c-Al_2_O_3_. Reaction conditions: GHSV = 7800 mL g^−1^ h^−1^, gas flow rate = 13 mL min^−1^, CO_2_:H_2_ = 1:3, P = 3.0 MPa.

The catalytic performances, including the CO_2_ conversion efficiency, selectivity towards methanol, and space–time yield for methanol formation, are presented in Figure 5. The influence of the temperature was examined in the range of 200 °C to 300 °C for the catalysts with CuZnO loadings from 3 to 20%, and the results are presented in Figure 5a. As expected, the CO_2_ conversion increased with the temperature for all of the examined catalysts because the thermodynamically stable CO_2_ molecule can be more efficiently broken down at high temperatures. However, at high reaction temperatures, the selectivity towards CH_3_OH formation presented in Figure 5b decreased, given the competitive advantage of the RWGS reaction, resulting in CO formation [36]. The increasing CuZnO loading increased the CH_3_OH selectivity from 58.5% for 3% CuZnO to 79.5% for 20% CuZnO at 200 °C, yet the improved selectivity did not essentially originate from increased CO_2_ conversion efficiency. The catalysts with 6–20% CuZnO had relatively similar CO_2_ conversion levels, higher than that of the catalyst containing 3% CuZnO by 12.6% at 300 °C. In addition, the methanol selectivity and STY of the former were higher than that of the latter at all temperatures (Figure 5b,c).

Since our aim was not to pursue a high efficiency for CO_2_ conversion to methanol but rather to explore and understand the influence of the catalyst structure on its catalytic performance, as well as the fact that the catalyst with 3% CuZnO showed a more homogeneous distribution of CuZnO on the catalyst support than the catalyst with higher loadings, we focused on the catalytic performance of the 3% CuZnO catalyst. As shown in Figure 5d–f, different samples containing 3% Cu, 3% ZnO, or 3% CuZnO were compared to better ascertain the role of each component and the synergistic effect. In addition, the catalytic performance of 3% CuZnO/n-Al_2_O_3_ was compared with that of 3% CuZnO/c-Al_2_O_3_ (commercial Al_2_O_3_). As shown in Figure 5a–f, improved catalytic performance was observed, with better CO_2_ conversion (19.8%) and *STY_CH3OH_* (1.31 mmol·g_cat_^−1^h^−1^) at 300 °C while utilizing the 3% CuZnO/n-Al_2_O_3_ sample. This combination outperformed those of the separate components, as well as the sample supported on the commercial Al_2_O_3._ However, the methanol selectivity on 3% CuZnO/n-Al_2_O_3_ was lower than those of the Cu/n-Al_2_O_3_ and CuZnO/c-Al_2_O_3_ at temperatures above 220 °C (Figure 5e). The 3% ZnO/n-Al_2_O_3_ exhibited the lowest catalytic activity under all studied temperatures. This observation was not unexpected because Cu is the main component that contributes to methanol selectivity. The pure n-Al_2_O_3_ support was found to have negligible activity for CH_3_OH production (data not shown). Overall, CuZnO exhibited better reactivity than either Cu or ZnO. While Cu is the methanol-selective component, ZnO and Al_2_O_3_ play other roles in the catalytic process [12].

#### 3.2.2. DRIFTS Study on the CO_2_ Activation

The CO_2_ activation was investigated via in situ DRIFT spectroscopy [37,38]. First, CO_2_ was adsorbed on the various fresh catalysts (at 250 °C, 2 MPa) that were reduced in H_2_ at 300 °C and the spectra were recorded. After sufficient adsorption of CO_2_ and attaining a stable state for up to 120 min, the inlet gas was changed to H_2_. The spectra were again recorded for up to 220 min under the same conditions. The different catalyst components were studied during the experiments, and their specific roles in CO_2_ activation and hydrogenation were elucidated.

(i) CO_2_ activation on n-Al_2_O_3_

**Figure 6:**
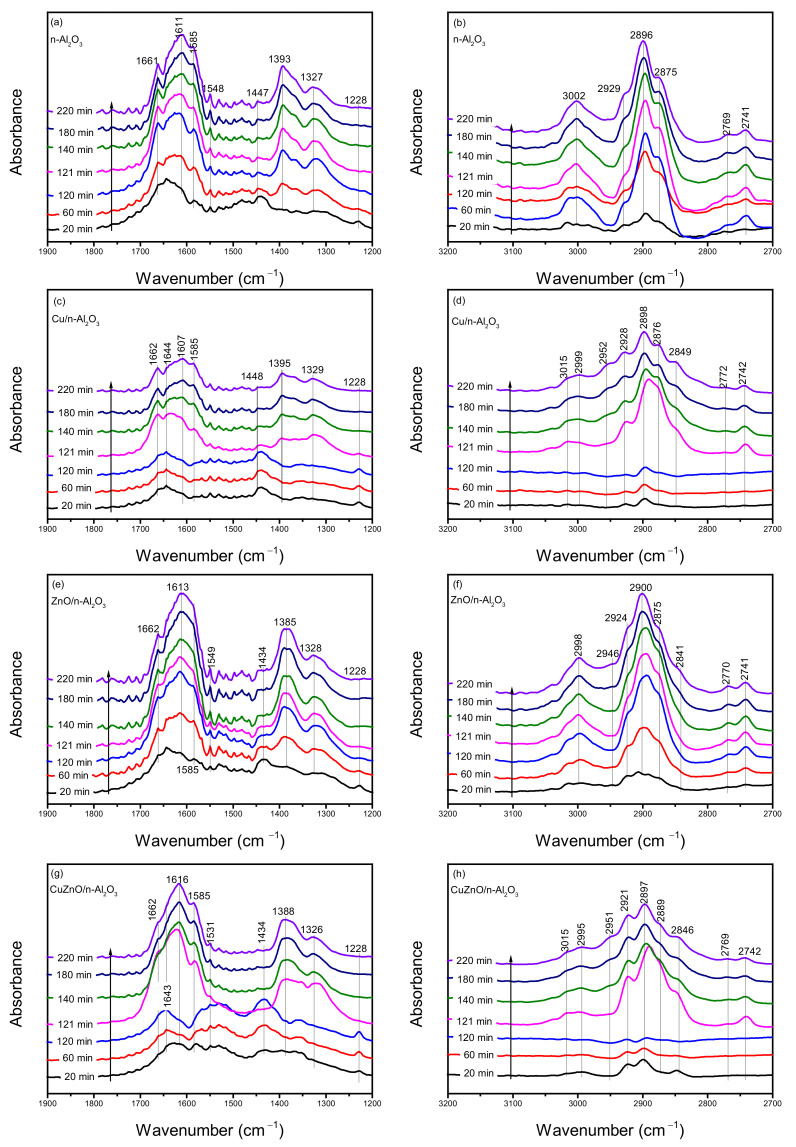
In situ DRIFT spectra at different times for the fresh catalysts (reduced in H_2_ at 300 °C, purged in N_2_, and then switched to CO_2_ gas): (**a**,**b**) n-Al_2_O_3_, (**c**,**d**) 3% Cu/n-Al_2_O_3_, (**e**,**f**) 3% ZnO/n-Al_2_O_3_, and (**g**,**h**) 3% CuZnO/n-Al_2_O_3_ at 250 °C under 2 MPa for both pure CO_2_ gas (0–120 min) and after switching the feed gas to H_2_ (120–220 min).

The pure n-Al_2_O_3_ in the CO_2_ stream evolves bands at 1661, 1611, 1585, 1548, 1447, 1393, 1327, and 1228 cm^−1^ attributed to carbonate species, indicating adsorption of CO_2_ on Al_2_O_3_ (Figure 6a). Specifically, the bands at 1661, 1585, and 1447 cm^−1^ are those of bicarbonates (HCO_3_^−^) species [39]. Other bands at 1548 and 1327 cm^−1^ can be assigned to polydentate carbonate, while the band at 1393 cm^−1^ belongs to monodentate carbonate. The 1228 cm^−1^ band can be attributed to the δ(OH) of bicarbonate species. This band decreases or even disappears with time. On the C-H stretching region in Figure 6b, bands can be observed at 3002, 2929, 2899-2896, 2875, 2769, and 2741 cm^−1^. According to the literature, the bands at 3002 and 2769 cm^−1^ were assigned to bidentate formate species on Al_2_O_3_-supported catalyst [40,41,42]; however, these bands were formed on pure alumina nanorods in this study. Here, we question the formation of formate species, since no H_2_ gas was passed through the sample during the first 120 min, except that certain reactions exist with OH groups on the Al_2_O_3_ surface [37]. Upon passing H_2_ gas through the sample, the band frequencies in the spectra did not change significantly, indicating that no substantial reaction occurred with the absorbed carbon species and pointing to the fact that Al_2_O_3_ cannot or can poorly activate H_2_. In addition, the presence of H_2_ may promote CO_2_ desorption from the Al_2_O_3_ surface, since there was no strong reaction between them (CO_2_ and Al_2_O_3_), as indicated by the disappearance of the band at 1228 cm^−1^. This result indicates that nanorod Al_2_O_3_ can be used as a support for the CO_2_ conversion catalyst due to its ability to adsorb CO_2_ in its activated forms.

(ii) CO_2_ activation on Cu/Al_2_O_3_ and ZnO/n-Al_2_O_3_

The formation of carbonate species on the surface of Cu/Al_2_O_3_ was obvious at 120 min, as evidenced by the bands at 1644, 1442, and 1228 cm^−1^ [39,42] (Figure 6c). Interestingly, these carbonate species disappeared and formate species formed after switching to H_2_. This indicates that Cu can activate H_2_ and promote its reaction with adsorbed CO_2_ species [43]. The formate species showed bands at 1662, 1585, 1395, and 1329 cm^−1^. These bands were attributed to bidentate formate species on both Cu and Al_2_O_3_ and could be confirmed by the new bands at 2999, 2952, and 2742 cm^−1^ in the C-H stretching spectra (Figure 6d) [41,44]. Wang et al. [37] assigned the band at around 2999 cm^−1^ to the C-H bonds in unsaturated CH*_x_* on CuNi catalysts, and its presence facilitated the deep hydrogenation of CO_2_ to form methanol. For ZnO/n-Al_2_O_3_ ((Figure 6e,f), after switching the feed gas from CO_2_ to H_2_, there was no strong indication of H_2_ activation; however, the appearance of bands of higher intensities and at nearly similar frequencies to that of pure n-Al_2_O_3_ is an indication that ZnO promoted the CO_2_ adsorption on the catalyst surface. The prominence of bands at all frequencies, which originated when only CO_2_ was adsorbed and maintained in the presence of H_2_, prove that ZnO activates CO_2_ mainly as carbonates. However, some bands exhibited slight shifts in their positions, which indicates some sort of interaction and formation of formate species. In a previous study, the bands at 1385 and 1328 cm^−1^ were assigned to bidentate formate on ZnO and Al_2_O_3_, whereas that at 1613 cm^−1^ was attributed to V_as_(OCO) of a bidentate carbonate species on ZnO [45]. The formation of formate species on ZnO can be confirmed by the evolution of 2946 and 2841 cm^−1^ bands in the C-H stretch spectra.

(iii) CO_2_ activation on CuZnO/Al_2_O_3_.

In the case of CuZnO/n-Al_2_O_3_, in the CO_2_ gas stream, similar spectral features to Cu/n-Al_2_O_3_ were observed. The peaks at 1643 and 1434 cm^−1^ were those of V_s_(OCO) of bicarbonate species [42]. The band at 1228 cm^−1^ was assigned to carboxylate (CO_2_^δ−^) species in previous studies [38,46], but we related the band to the δ(OH) of bicarbonate species, as earlier mentioned. However, the peak intensities were found to be higher than those obtained from Cu/n-Al_2_O_3_. This was attributed to the contribution of ZnO, which promotes CO_2_ adsorption. After switching the gas to H_2_, most of the formed carbonate species converted into the formate species by reacting with H-atoms. This was evident from analyzing the bands at 1616, 1585, 1388, and 1326 cm^−1^, which indicated formate species on Al_2_O_3_, ZnO, and Cu [41,45]. The increased band intensities at 2897, 2921, and 2995 cm^−1^ indicated that the evolution of bidentate formate on the catalyst increased with time. It follows that CuZnO/n-Al_2_O_3_ exhibited the best CO_2_ activation, good H_2_ activation, and catalytic activity for converting carbonate into formate, which is an important intermediate for methanol production. The increase in peak intensity means that the CO_2_ adsorption intensified with time and reached a maximum (steady-state) at 120 min.

#### 3.2.3. DRIFTS Study of Methanol Formation from CO_2_ Hydrogenation

**Figure 7:**
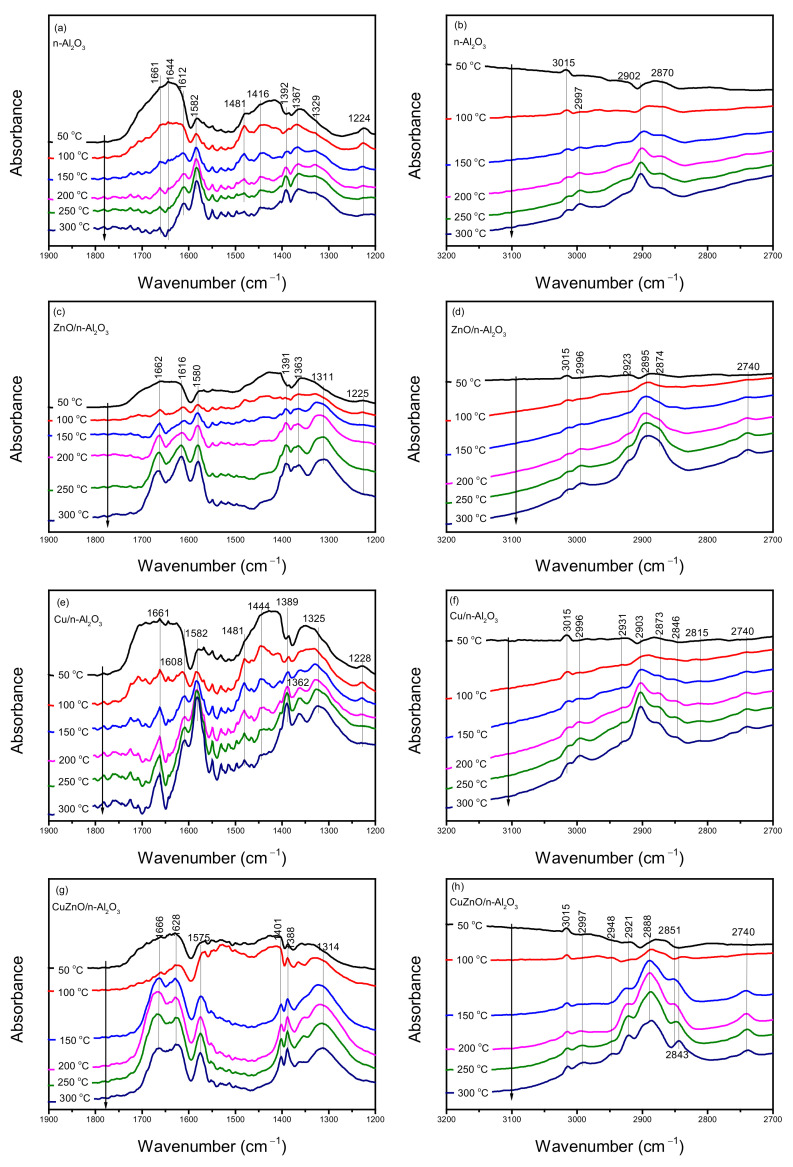
In situ DRIFT spectra of the reaction gas mixture of (**a**,**b**) n-Al_2_O_3_, (**c**,**d**) 3% ZnO/n-Al_2_O_3_, (**e**,**f**) 3% Cu/n-Al_2_O_3_, and (**g**,**h**) 3% CuZnO/n-Al_2_O_3_. Reaction conditions: CO_2_:H_2_ = 1:3, gas flow rate = 30 mL min^−1^, P = 2.0 MPa.

The key surface species and intermediates involved in forming methanol over the catalysts are shown in Figure 7. The in situ DRIFT spectra collected at different temperatures on n-Al_2_O_3_, ZnO/Al_2_O_3_, Cu/n-Al_2_O_3_, and CuZnO/n-Al_2_O_3_ dosed with the reaction gas mixture indicated that the hydrogenation of CO_2_ was initiated at a temperature above 50 °C. The initiation temperature decreases when the catalyst contains an active phase (Figure 7d,f,h). Except for the band around 1228 cm^−1^, which is associated with δ(OH) of bicarbonate species or weakly adsorbed carbonate species (CO_3_^2−^) that are desorbed at high temperatures, there is an increase in the intensities of the bands of hydrogenated species as the temperature rises. This means that the formation of key surface species increases with the temperature. The peaks at 1644 and 1416 cm^−1^ are attributed to bicarbonate species formed when the reaction gas mixture passed through n-Al_2_O_3_ at 50 °C (Figure 7a). At higher temperatures, formate species formation is evident, typically indicated by the bands at 1612, 1583, 1391, and 1329 cm^−1^. The formate species are also confirmed with the C-H stretching bands at 2997 and 2902 cm^−1^ in Figure 7b, which are identical to those observed when formic acid is adsorbed and evacuated at 473 K on Al_2_O_3_ [41]. In Figure 7e,f, the bands at 1661_,_ 1608, 1582, 1389, and 1325 cm^−1^ are attributed to formate species on Cu and Al_2_O_3_ [40]. Specifically, the band at 1325 cm^−1^ is for V_s_(OCO) of bidentate formate species on Cu [40,41]. Other bands also evolved at 2931 and 2740 cm^−1^, related to v(C-H)+v_as_(OCO) and v(C-H)+v_s_(OCO) of bidentate formate on Al_2_O_3_ [40]. On CuZnO/n-Al_2_O_3_ (Figure 7g,h), the reaction gas is adsorbed as carbonate species up to 100 °C, as indicated by the adsorption bands around 1526 and 1415 cm^−1^ for polydentate carbonate and bicarbonate species, respectively [40]. The formation of formate species can be related to the bands at 1666 and 1628 cm^−1^. The bidentate formate species on ZnO and Al_2_O_3_ showed bands of higher intensities at 1575, 1388, 1314, 2888, and 2740 cm^−1^ [41,45]. Meanwhile, the 2921 cm^−1^ band can be assigned to the v(C-H) and δ(C-H)+v_as_(OCO) of bidentate formate on Cu [41,44]. The peaks at 2948 and 2843 in the CuZnO/n-Al_2_O_3_ spectra and at 2931 and 2846 in the Cu/n-Al_2_O_3_ spectra indicate the formation of methoxy species [30]. At 150 °C or higher temperatures, the band intensities increased compared with those of Cu/n-Al_2_O_3_. For these catalysts, the carbonate conversion to formate species is seen on both CuZnO and Cu, but the formation of formate species begins at a low temperature (100 °C) exclusively on Cu/n-Al_2_O_3_. The key intermediate species formed at different temperatures are summarized in Table 2. The introduction of ZnO enhances the CO_2_ adsorption and facilitates more carbonate species formation. This, in turn, boosts the formate production through hydrogenation via dissociated H atoms, which spillover to the catalyst surface. The formation of formate at a lower temperature on the CuZnO/n-Al_2_O_3_ catalyst might be ascribed to a more effective H_2_ dissociation in the presence of ZnO [40]. Generally, two reaction pathways for the hydrogenation of the CO_2_ to methanol have been identified: RWGS + CO-hydro, along with formate pathways [47]. However, many studies on Cu-based catalysts conform to the formate pathway [30,38]. In this study, CO_2_ is activated as carbonate species on the catalyst surface under the examined reaction conditions. As the temperature increases, H_2_ is better activated and spills over to the catalyst surface, where it reacts with the carbonate and forms formate and methoxy species. Further hydrogenation of the surface species produces methanol. This mechanism is, thus, in good agreement with literature reports for other Cu-based systems.

### 3.3. Roles of Al_2_O_3_, ZnO, and Cu in CO_2_ Hydrogenation to Methanol

**Scheme 1:**
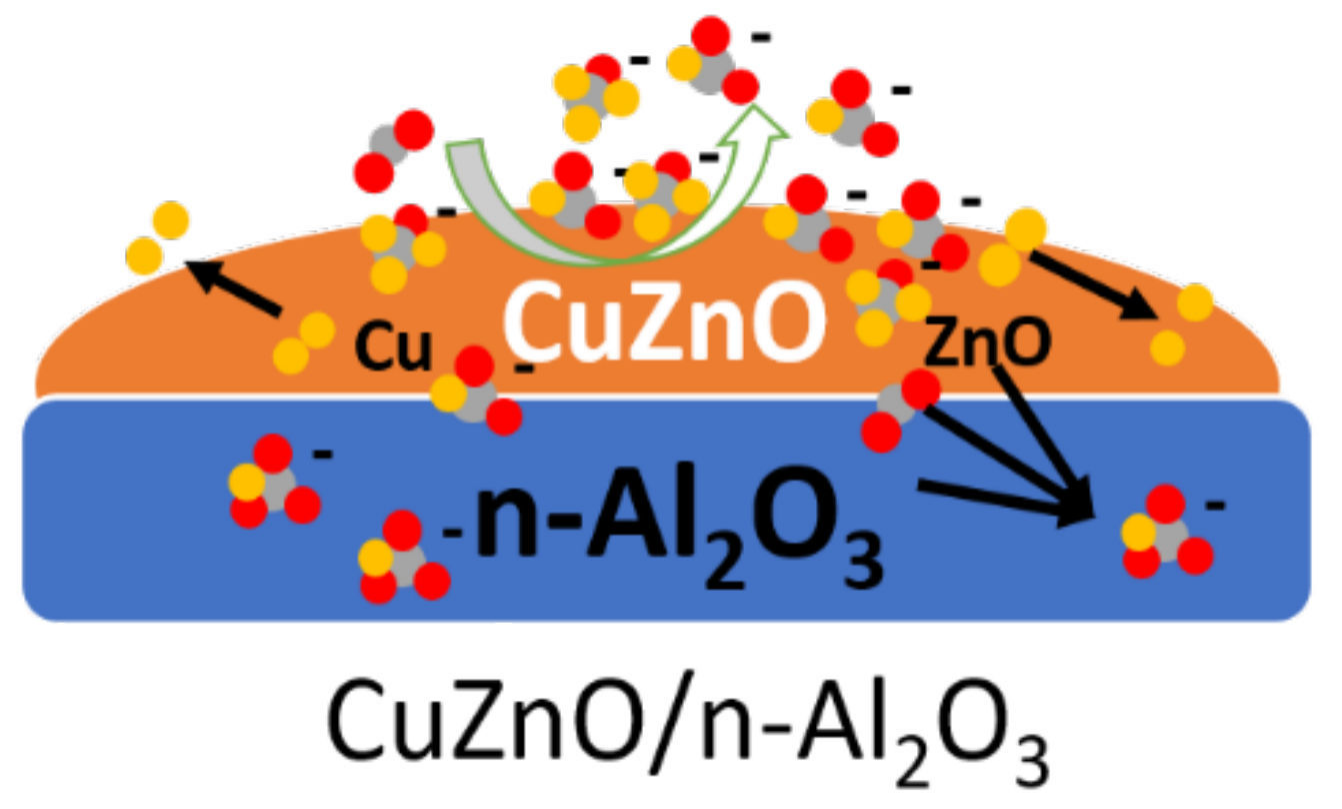
Formation of key intermediate species in hydrogenation of CO_2_ to methanol (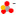 bicarbonate, 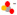 formate, 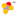 methoxy, 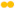 hydrogen).

Understanding the structure–function relationship of a composite catalyst is important to develop more efficient catalysts for industrial applications. The individual roles of Al_2_O_3_, ZnO, and Cu and their synergistic effects in the CuZnO/Al_2_O_3_ catalyst used for the CO_2_-to-methanol synthesis were investigated using DRIFT spectroscopy. Al_2_O_3_ is a large surface area support material with an appropriate surface for dispersing and stabilizing the active phase and slowing the sintering during the reaction at high temperatures [40]. Herein, we found Al_2_O_3_ to be a good material for CO_2_ adsorption and activation to produce bicarbonate, carbonate, and formate species; the bicarbonate and carbonate species are converted into formate and methoxy in the presence of metal species that can activate H_2_. The 3% Cu/n-Al_2_O_3_ and 3% CuZnO/n-Al_2_O_3_ catalysts possess nearly similar BET surface areas of 94.4 and 89.4 m^2^/g, respectively. This shows that both active phases had the same impact on the surface area; however, the catalysts exhibit slight differences in their CO_2_ adsorption and catalytic activities. Methoxy and increasing amounts of formate species are produced for both catalysts. The conversion of carbonates to formate is evident, as H-atoms are available for the reaction after activation on Cu particles following the spillover effect. According to the catalytic performance and in situ DRIFT results, CuZnO/n-Al_2_O_3_ exhibited the best performance due to the synergistic effect resulting from the interaction of Cu and ZnO. Apparently, as ascertained from the in situ DRIFT spectra, the presence of ZnO with Cu promotes CO_2_ adsorption and formate formation. The enhanced formate formation is thought to be due to additional H_2_ dissociation activity. We, thus, suggest that ZnO also promotes H_2_ activation. It was previously reported that a defective ZnO_x_ overlayer formed in a Cu/ZnO catalyst could dissociate H_2_, even at room temperature [40]. From the above discussion, it will be reasonable to conclude from our study that the different components of the industrial catalyst for methanol synthesis from CO_2_ play different roles but with a cooperative effect in methanol formation, as shown in Scheme 1.

## 4. Conclusions

Cu-based catalysts supported on Al_2_O_3_ nanorods for CO_2_ activation and subsequent hydrogenation to methanol are discussed in this study. Compared with the commercial Al_2_O_3_ support, catalysts using Al_2_O_3_ nanorods as the support show higher efficiency for CO_2_ conversion between 200 and 300 °C, but the methanol selectivity becomes lower above 220 °C. There is improved CO_2_ adsorption on the Al_2_O_3_ nanorods. CO_2_ is adsorbed and activated on the catalyst surface as carbonate species, which upon subsequent H_2_ dissociation on Cu hydrogenates to formate species by reacting with the H atom. ZnO promotes CO_2_ adsorption and probably facilitates H_2_ dissociation on Cu, also leading to the more efficient hydrogenation of activated CO_2_ species on CuZnO/Al_2_O_3_. This study will help to tailor the properties and composition of the Cu-based catalyst system to achieve higher efficiency for methanol synthesis from CO_2_.

## Figures and Tables

**Table 1:** EDX analysis of the 3% CuZnO/n-Al_2_O_3_ catalyst.

Element	Cu	Zn	Al	O
(At%)	0.81	0.29	40.57	58.33
(Wt%)	2.46	0.88	52.18	44.48

**Table 2:** Formation of the key intermediates and their peak intensity changes with temperature and catalyst composition *.

Temperature (°C)	n-Al_2_O_3_	3% ZnO/n-Al_2_O_3_	3% Cu/n-Al_2_O_3_	3% CuZnO/n-Al_2_O_3_
50	Bicarbonate	Bicarbonate (ZnO), bicarbonate (Al_2_O_3_)	Bicarbonate (Al_2_O_3_)	Bicarbonate (ZnO)
100	Bicarbonate ↓, monodentate, carbonate bidentate formate	Bidentate carbonate (ZnO) ↓, bicarbonate (Al_2_O_3_) ↓, polydentate carbonate (Al_2_O_3_ or ZnO), bidentate formate (ZnO and Al_2_O_3_)	Bicarbonate (Al_2_O_3_) ↓, bidentate formate (Al_2_O_3_), polydentate carbonate	Bicarbonate (ZnO) ↓, polydentate carbonate (Al_2_O_3_)
150	Bicarbonate ↓, polydentate carbonate, bidentate formate ↑	Bidendentate formate (ZnO and Al_2_O_3_) ↑, monodentate carbonate (Al_2_O_3_) ↓, bicarbonate (Al_2_O_3_) ↓, polydentate carbonate (Al_2_O_3_) ↑	Bicarbonate (Al_2_O_3_) ↓, bidentate formate (Cu), bidendentate formate (Al_2_O_3_) ↑	Bicarbonate (ZnO) ↑, polydentate carbonate (Al_2_O_3_), bidentate formate (Al_2_O_3_ and ZnO), bidentate formate (Cu), bidendate formate (ZnO) ↑, methoxy
200	Bicarbonate ↓, monodentate carbonate ↓, bidentate formate ↑	Bicarbonate (ZnO) ↑, bidentate carbonate (ZnO) ↑, bidentate formate (ZnO and Al_2_O_3_) ↑, polydentate carbonate (ZnO) ↑	Bicarbonate (Al_2_O_3_) ↓, bidentate formate (Cu), bidentate formate (Al_2_O_3_) ↑, methoxy	Bidentate formate (Al_2_O_3_ and ZnO) ↑, bidentate formate (Cu) ↑, bidentate formate (ZnO) ↑, bidentate formate (Al_2_O_3_), methoxy
250	Bicarbonate ↓, nonodentate carbonate ↓, polydentate carbonate ↓, bidentate formate ↑	Bicarbonate (ZnO) ↑, bidentate carbonate (ZnO) ↑, bidentate formate (ZnO and Al_2_O_3_) ↑, formate (Al_2_O_3_ ↑)	Bidendate formate (Cu) ↑, bidentate formate (Al_2_O_3_) ↑, methoxy ↑	Bidentate formate (Al_2_O_3_ and ZnO) ↑, bidentate formate (Cu) ↑, bidentate formate (ZnO) ↑, bidentate formate (Al_2_O_3_) ↑, methoxy ↑
300	Same as in 250 °C	Bicarbonate (ZnO) ↑, bidentate carbonate (ZnO) ↑, bidentate formate (ZnO and Al_2_O_3_) ↑, formate (Al_2_O_3_) ↑	Same as in 250°C	Bidendate formate (Al_2_O_3_ and ZnO) ↑, bidentate formate (Cu) ↑, Bidentate formate (ZnO) ↑, bidentate formate (Al_2_O_3_) ↑, methoxy ↑

* ↓—Decrease; ↑—increase.

## References

[1] Porosoff M.D., Yan B., Chen J.G. (2016). Catalytic reduction of CO_2_ by H_2_ for synthesis of CO, methanol and hydrocarbons: Challenges and opportunities. Energy Environ. Sci..

[2] Wang Q., Luo J., Zhong Z., Borgna A. (2011). CO_2_ capture by solid adsorbents and their applications: Current status and new trends. Energy Environ. Sci..

[3] Wang J., Huang L., Yang R., Zhang Z., Wu J., Gao Y., Wang Q., O’Hare D., Zhong Z. (2014). Recent advances in solid sorbents for CO_2_ capture and new development trends. Energy Environ. Sci..

[4] Aresta M., Dibenedetto A., Angelini A. (2014). Catalysis for the Valorization of Exhaust Carbon: From CO_2_ to Chemicals, Materials, and Fuels. Technological Use of CO_2_. Chem. Rev..

[5] Porosoff M.D., Yang X., Boscoboinik J.A., Chen J.G. (2014). Molybdenum Carbide as Alternative Catalysts to Precious Metals for Highly Selective Reduction of CO_2_ to CO. Angew. Chem. Int. Ed..

[6] Studt F., Sharafutdinov I., Abild-Pedersen F., Elkjær C.F., Hummelshøj J.S., Dahl S., Chorkendorff I., Nørskov J.K. (2014). Discovery of a Ni-Ga catalyst for carbon dioxide reduction to methanol. Nat. Chem..

[7] Sehested J. (2019). Industrial and scientific directions of methanol catalyst development. J. Catal..

[8] De S., Dokania A., Ramirez A., Gascon J. (2020). Advances in the Design of Heterogeneous Catalysts and Thermocatalytic Processes for CO_2_ Utilization. ACS Catal..

[9] Kattel S., Liu P., Chen J.G. (2017). Tuning selectivity of CO_2_ hydrogenation reactions at the metal/oxide interface. J. Am. Chem. Soc..

[10] Wang J., Zhang G., Zhu J., Zhang X., Ding F., Zhang A., Guo X., Song C. (2021). CO_2_ hydrogenation to methanol over In_2_O_3_-based catalysts: From mechanism to catalyst development. ACS Catal..

[11] Lin L., Wang G., Zhao F. (2021). CO_2_ Hydrogenation to Methanol on ZnO/ZrO_2_ Catalysts: Effects of Zirconia Phase. ChemistrySelect.

[12] Etim U., Song Y., Zhong Z. (2020). Improving the Cu/ZnO-Based Catalysts for Carbon Dioxide Hydrogenation to Methanol, and the Use of Methanol as a Renewable Energy Storage Media. Front. Energy Res..

[13] Etim U.J., Semiat R., Zhong Z. (2021). CO_2_ Valorization Reactions over Cu-Based Catalysts: Characterization and the Nature of Active Sites. Am. J. Chem. Eng..

[14] Dong X., Li F., Zhao N., Xiao F., Wang J., Tan Y. (2016). CO_2_ hydrogenation to methanol over Cu/ZnO/ZrO_2_ catalysts prepared by precipitation-reduction method. Appl. Catal. B Environ..

[15] Natesakhawat S., Lekse J.W., Baltrus J.P., Ohodnicki P.R., Howard B.H., Deng X., Matranga C. (2012). Active sites and structure–activity relationships of copper-based catalysts for carbon dioxide hydrogenation to methanol. ACS Catal..

[16] Huš M., Kopač D., Štefančič N.S., Jurković D.L., Dasireddy V.D., Likozar B. (2017). Unravelling the mechanisms of CO_2_ hydrogenation to methanol on Cu-based catalysts using first-principles multiscale modelling and experiments. Catal. Sci. Technol..

[17] Kuld S., Thorhauge M., Falsig H., Elkjær C.F., Helveg S., Chorkendorff I., Sehested J. (2016). Quantifying the promotion of Cu catalysts by ZnO for methanol synthesis. Science.

[18] Nitta Y., Suwata O., Ikeda Y., Okamoto Y., Imanaka T. (1994). Copper-zirconia catalysts for methanol synthesis from carbon dioxide: Effect of ZnO addition to Cu-ZrO_2_ catalysts. Catal. Lett..

[19] An B., Zhang J., Cheng K., Ji P., Wang C., Lin W. (2017). Confinement of Ultrasmall Cu/ZnO_x_ Nanoparticles in Metal–Organic Frameworks for Selective Methanol Synthesis from Catalytic Hydrogenation of CO_2_. J. Am. Chem. Soc..

[20] Dasireddy V.D., Likozar B. (2019). The role of copper oxidation state in Cu/ZnO/Al_2_O_3_ catalysts in CO_2_ hydrogenation and methanol productivity. Renew. Energ..

[21] Behrens M., Studt F., Kasatkin I., Kühl S., Hävecker M., Abild-Pedersen F., Zander S., Girgsdies F., Kurr P., Kniep B.-L. (2012). The active site of methanol synthesis over Cu/ZnO/Al_2_O_3_ industrial catalysts. Science.

[22] Liang B., Ma J., Su X., Yang C., Duan H., Zhou H., Deng S., Li L., Huang Y. (2019). Investigation on Deactivation of Cu/ZnO/Al_2_O_3_ Catalyst for CO_2_ Hydrogenation to Methanol. Ind. Eng. Chem. Res..

[23] Narayanan R., El-Sayed M.A. (2005). Catalysis with Transition Metal Nanoparticles in Colloidal Solution: Nanoparticle Shape Dependence and Stability.

[24] Sakpal T., Lefferts L. (2018). Structure-dependent activity of CeO_2_ supported Ru catalysts for CO_2_ methanation. J. Catal..

[25] Lei H., Nie R., Wu G., Hou Z. (2015). Hydrogenation of CO_2_ to CH_3_OH over Cu/ZnO catalysts with different ZnO morphology. Fuel.

[26] Liao F., Huang Y., Ge J., Zheng W., Tedsree K., Collier P., Hong X., Tsang S.C. (2011). Morphology—Dependent Interactions of ZnO with Cu Nanoparticles at the Materials’ Interface in Selective Hydrogenation of CO_2_ to CH_3_OH. Angew. Chem. Int. Ed..

[27] An X., Li J., Zuo Y., Zhang Q., Wang D., Wang J. (2007). A Cu/Zn/Al/Zr fibrous catalyst that is an improved CO_2_ hydrogenation to methanol catalyst. Catal. Lett..

[28] Huttunen P.K., Labadini D., Hafiz S.S., Gokalp S., Wolff E.P., Martell S.M., Foster M. (2021). DRIFTS investigation of methanol oxidation on CeO_2_ nanoparticles. Appl. Surf. Sci..

[29] Reddy K.P., Choi H., Kim D., Choi M., Ryoo R., Park J.Y. (2021). The facet effect of ceria nanoparticles on platinum dispersion and catalytic activity of methanol partial oxidation. Chem Commun.

[30] Chen K., Fang H., Wu S., Liu X., Zheng J., Zhou S., Duan X., Zhuang Y., Tsang S.C.E., Yuan Y. (2019). CO_2_ hydrogenation to methanol over Cu catalysts supported on La-modified SBA-15: The crucial role of Cu–LaOx interfaces. Appl. Catal. B Environ..

[31] Zhu J., Su Y., Chai J., Muravev V., Kosinov N., Hensen E.J. (2020). Mechanism and nature of active sites for methanol synthesis from CO/CO_2_ on Cu/CeO_2_. ACS Catal..

[32] Shen S., Chen Q., Chow P., Tan G., Zeng X., Wang Z., Tan R.B. (2007). Steam-assisted solid wet-gel synthesis of high-quality nanorods of boehmite and alumina. J. Phy. Chem. C.

[33] Bai P., Su F., Wu P., Wang L., Lee F.Y., Lv L., Yan Z.-f., Zhao X.S. (2007). Copolymer-Controlled Homogeneous Precipitation for the Synthesis of Porous Microfibers of Alumina. Langmuir.

[34] Martínez A., Prieto G., Rollán J. (2009). Nanofibrous γ-Al_2_O_3_ as support for Co-based Fischer–Tropsch catalysts: Pondering the relevance of diffusional and dispersion effects on catalytic performance. J. Catal..

[35] Thommes M., Kaneko K., Neimark A.V., Olivier J.P., Rodriguez-Reinoso F., Rouquerol J., Sing K.S. (2015). Physisorption of gases, with special reference to the evaluation of surface area and pore size distribution (IUPAC Technical Report). Pure Appl. Chem..

[36] Zhang C., Wang L., Etim U.J., Song Y., Gazit O.M., Zhong Z. (2022). Oxygen vacancies in Cu/TiO_2_ boost strong metal-support interaction and CO_2_ hydrogenation to methanol. J. Catal..

[37] Wang L.-X., Guan E., Wang Z., Wang L., Gong Z., Cui Y., Yang Z., Wang C., Zhang J., Meng X. (2020). Dispersed nickel boosts catalysis by copper in CO_2_ hydrogenation. ACS Catal..

[38] Kattel S., Yan B., Yang Y., Chen J.G., Liu P. (2016). Optimizing binding energies of key intermediates for CO_2_ hydrogenation to methanol over oxide-supported copper. J. Am. Chem. Soc..

[39] Bansode A., Tidona B., von Rohr P.R., Urakawa A. (2013). Impact of K and Ba promoters on CO_2_ hydrogenation over Cu/Al_2_O_3_ catalysts at high pressure. Catal. Sci. Technol..

[40] Hu J., Li Y., Zhen Y., Chen M., Wan H. (2021). In situ FTIR and ex situ XPS/HS-LEIS study of supported Cu/Al_2_O_3_ and Cu/ZnO catalysts for CO_2_ hydrogenation. Chin. J. Catal..

[41] Bando K.K., Sayama K., Kusama H., Okabe K., Arakawa H. (1997). In-situ FT-IR study on CO_2_ hydrogenation over Cu catalysts supported on SiO_2_, Al_2_O, and TiO_2_. Appl. Catal. A Gen..

[42] Baltrusaitis J., Jensen J.H., Grassian V.H. (2006). FTIR spectroscopy combined with isotope labeling and quantum chemical calculations to investigate adsorbed bicarbonate formation following reaction of carbon dioxide with surface hydroxyl groups on Fe_2_O_3_ and Al_2_O_3_. J. Phys. Chem. B.

[43] Sakong S., Groß A. (2003). Dissociative adsorption of hydrogen on strained Cu surfaces. Surf. Sci..

[44] Fujita S.-I., Usui M., Ohara E., Takezawa N. (1992). Methanol synthesis from carbon dioxide at atmospheric pressure over Cu/ZnO catalyst. Role of methoxide species formed on ZnO support. Catal. Lett..

[45] Bailey S., Froment G., Snoeck J.-W., Waugh K. (1994). A DRIFTS study of the morphology and surface adsorbate composition of an operating methanol synthesis catalyst. Catal. Lett..

[46] Kim Y., Trung T.S.B., Yang S., Kim S., Lee H. (2016). Mechanism of the surface hydrogen induced conversion of CO_2_ to methanol at Cu (111) step sites. ACS Catal..

[47] Liu C., Liu P. (2015). Mechanistic study of methanol synthesis from CO_2_ and H_2_ on a modified model Mo_6_S_8_ cluster. ACS Catal..

